# Remnant Cholesterol Levels at Diagnosis May Predict Acute Coronary Syndrome Occurrence During Follow-Up in Patients with Antineutrophil Cytoplasmic Antibody-Associated Vasculitis

**DOI:** 10.3390/jcm14072260

**Published:** 2025-03-26

**Authors:** Hyunsue Do, Oh Chan Kwon, Jang Woo Ha, Jihye Chung, Yong-Beom Park, Ji Hye Huh, Sang-Won Lee

**Affiliations:** 1 Division of Rheumatology, Department of Internal Medicine, Kangwon National University School of Medicine, Chuncheon-si 24341, Republic of Korea; dohysu@naver.com; 2 Division of Rheumatology, Department of Internal Medicine, Gangnam Severance Hospital, Yonsei University College of Medicine, Seoul 06273, Republic of Korea; ockwon@yuhs.ac; 3 Division of Rheumatology, Department of Internal Medicine, Yongin Severance Hospital, Yonsei University College of Medicine, Yongin 16995, Republic of Korea; hjwnmk@yuhs.ac; 4 Division of Rheumatology, Department of Internal Medicine, Severance Hospital, Yonsei University College of Medicine, Seoul 03722, Republic of Korea; jchung0831@yuhs.ac (J.C.); yongbpark@yuhs.ac (Y.-B.P.); 5 Institute for Immunology and Immunological Diseases, Yonsei University College of Medicine, Seoul 03722, Republic of Korea; 6 Division of Endocrinology and Metabolism, Department of Internal Medicine, Hallym University Sacred Heart Hospital, Anyang 14068, Republic of Korea

**Keywords:** remnant cholesterol, antineutrophil cytoplasmic antibody, vasculitis, acute coronary syndrome, predict

## Abstract

**Background/Objectives**: Previous studies have revealed the predictive potential of remnant cholesterol (RC) for acute coronary syndrome (ACS) occurrence in the general population. However, whether this association applies to patients with antineutrophil cytoplasmic antibody-associated vasculitis (AAV), in which a lipid paradox exists, remains unclear. We investigated whether RC levels at diagnosis could predict ACS occurrence during follow-up in patients with AAV. **Methods**: This study included 139 patients with AAV. ACS was defined as ST-elevation myocardial infarction (STEMI), non-STEMI, or unstable angina occurring after AAV diagnosis. RC levels were calculated as (total cholesterol)–(low-density lipoprotein cholesterol)–(high-density lipoprotein cholesterol). Patients were categorised into three groups by RC tertiles: highest (≥26.2 mg/dL), middle (19.1−26.1 mg/dL), and lowest (≤19.0 mg/dL) tertile groups. **Results**: The median age of the 139 patients (male, 31.7%) was 58.0 years. During follow-up, six, two, and one patients were diagnosed with ACS in the highest, middle, and lowest tertile groups, respectively. Patients in the highest tertile group exhibited a significantly lower ACS-free survival rate than those in the lowest tertile (*p* = 0.030). In the multivariable Cox hazards model, male sex (hazard ratio [HR] 9.054, 95% confidence interval [CI] 1.786−45.910), Birmingham vasculitis activity score (HR 1.147, 95% CI 1.033−1.274), and the highest tertile of RC levels (HR 10.818, 95% CI 1.867–62.689) were significantly and independently associated with ACS occurrence during follow-up in patients with AAV. **Conclusions**: Our findings indicate that RC levels at diagnosis may predict ACS occurrence during follow-up in patients with AAV, regardless of the traditional cardiovascular risk factors.

## 1. Introduction

Antineutrophil cytoplasmic antibody (ANCA)-associated vasculitis (AAV) is a type of small-vessel vasculitis along with immune complex vasculitis [1]. According to clinical, laboratory, radiological, and histological features, AAV is categorised into three subtypes, including microscopic polyangiitis (MPA), granulomatosis with polyangiitis (GPA), and eosinophilic GPA (EGPA) [1,2,3,4,5]. Cardiovascular diseases (CVDs) are associated with various chronic inflammatory conditions [6]. These associations may also apply to patients with AAV. A recent meta-analysis showed that the risk of all CVD events in patients with AAV was approximately 65% higher than that in the general public [7]. Therefore, to manage CVD occurrence, the need for a serum biomarker to predict acute coronary syndrome (ACS), including ST-elevation myocardial infarction (STEMI), non-STEMI, and unstable angina after AAV diagnosis, has been suggested [8].

Atherogenesis is a chronic progressive disease mainly driven by lipids. Studies have shown that dyslipidaemia is majorly involved in the occurrence and progression of atherosclerotic CVD [9]. To date, lipid-lowering agents targeting low-density lipoprotein cholesterol (LDL-C), such as statins, have been the first-line therapeutic regimens for CVD and have contributed to reducing CVD-related mortality rates for decades [10]. Nevertheless, substantial residual risks for CVD persist, and the majority of predicted initial and recurrent CVD events have not been averted [11,12]. Recent studies suggest that circulating lipoprotein components other than LDL-C may also play crucial roles in CVD occurrence [13,14]. Triglyceride (TG)-rich lipoproteins and remnant cholesterol (RC), found in intermediate-density lipoprotein and very-low-density lipoprotein particles, are currently recognised as emerging risk factors for CVD [15,16]. To date, many studies have revealed the predictive potential of RC for CVD occurrence in the general population; however, no study has investigated its potential in patients with AAV [17]. Importantly, the lipid paradox has been reported in autoimmune rheumatic diseases [18,19]. Lower total cholesterol and LDL-C levels are associated with an increased risk of CVD in patients with autoimmune rheumatic diseases [18,19]. Therefore, it is unclear whether the association between RC levels and CVD occurrence observed in the general population also applies to patients with AAV. Here, we aimed to investigate whether RC levels at diagnosis could predict ACS occurrence during follow-up in patients with immunosuppressive drug-naive AAV.

## 2. Materials and Methods

### 2.1. Patients

This study selected 139 immunosuppressive drug-naïve patients with AAV from the Severance Hospital ANCA-associated Vasculitides (SHAVE) cohort, an observational single-centre cohort, based on the following inclusion and exclusion criteria:

#### 2.1.1. Inclusion Criteria

- Diagnosis with AAV for the first time at the Division of Rheumatology, Department of Internal Medicine, Yonsei University College of Medicine, Severance Hospital.
- Fulfilment of the 2007 European Medicines Agency algorithm for AAV, the 2012 revised Chapel Hill Consensus Conference nomenclature of vasculitides, and the 2022 American College of Rheumatology/European Alliance of Associations for Rheumatology classification criteria for MPA, GPA, and EGPA [1,2,3,4,5].
- Complete medical records, including information on clinical, laboratory, radiological, and histological data at diagnosis, as well as poor outcomes during follow-up.
- Followed up for a minimum of 3 months.

#### 2.1.2. Exclusion Criteria

- Having serious medical conditions that mimic AAV or induce false ANCA positivity, such as malignancies, infectious diseases requiring hospitalisation, and other systemic autoimmune diseases at the time of AAV diagnosis.
- Receiving glucocorticoids (≥20 mg/day equivalent to prednisolone) or immunosuppressive drugs within 1 month before AAV diagnosis.

This study was approved by the Institutional Review Board (IRB) of Severance Hospital (Seoul, Republic of Korea, IRB No. 4-2020-1071). The requirement for additional written informed consent was waived by the IRB owing to the retrospective nature of this study and the use of anonymised patient data.

### 2.2. Clinical Data

Regarding the variables during AAV diagnosis, age, sex, body mass index (BMI), and smoking history (no current smoker) were collected as demographic data. AAV subtype, ANCA positivity, and AAV-specific indices, including the Birmingham vasculitis activity score (BVAS) and five-factor score (FFS), were obtained [20,21]. In terms of ANCA measurement, perinuclear (P)-ANCAs and cytoplasmic (C)-ANCAs were detected using indirect immunofluorescence assays, whereas myeloperoxidase (MPO)-ANCAs and proteinase 3 (PR3)-ANCAs were determined using immunoassays. In this study, all four types of ANCAs were considered indicative of ANCA positivity [3,4,5,22]. Laboratory data included erythrocyte sedimentation rate (ESR) and C-reactive protein (CRP), total cholesterol, high-density lipoprotein cholesterol (HDL-C), and TG levels. Hypertension and type 2 diabetes mellitus (T2DM) were assessed as comorbidities.

Regarding the variables during follow-up, all-cause mortality, cerebrovascular accidents (CVAs), and ACS were investigated as poor outcomes of AAV related to dyslipidaemia. ACS included STEMI, non-STEMI, and unstable angina [8]. All-cause mortality, CVAs, and ACS were considered poor outcomes only when they occurred after AAV diagnosis. The occurrence of poor outcomes was determined through a retrospective review of medical records up to the patient’s last visit date. The follow-up duration was from the date of AAV diagnosis to either the date of the last visit or the date of poor outcome occurrence, whichever occurred first.

### 2.3. Measurements of the Lipid Profile

Blood samples were collected after an overnight fast. Lipid profiles, including total cholesterol, TG, and HDL-C levels, were measured using enzymatic methods. LDL-C levels were calculated using the Friedewald formula: LDL-C = total cholesterol minus HDL-C minus TG divided by 5, unless TG level was significantly elevated (>4.5 mmol/L or 400 mg/dL) [23]. RC levels are usually calculated as total cholesterol minus LDL-C minus HDL-C levels [24].

### 2.4. Tertiles According to RC Levels

Patients with AAV in this study were categorised into three groups by RC tertiles (values at 33.3 [19.0 mg/dL] and 66.6 [26.2 mg] percentiles, such as the highest, middle, and lowest tertiles). The number of patients belonging to the highest (≥26.2 mg/dL), middle (19.1−26.1 mg/dL), and lowest (≤19.0 mg/dL) tertile groups were 47, 45, and 47, respectively.

### 2.5. Statistical Analyses

All statistical analyses were performed using IBM SPSS Statistics for Windows version 26 (IBM Corp., Armonk, NY, USA). Continuous variables are expressed as medians with 25–75 percentiles, whereas categorical variables are expressed as numbers (percentages). The statistically significant area under the curve (AUC) of RC levels for each poor outcome was confirmed, and the optimal cutoff was extrapolated by performing a receiver operating characteristic (ROC) curve analysis. Significant differences among more than two categorical variables were analysed using the analysis of variance (ANOVA). The relative risk (RR) of ACS between those with RC levels ≥ cut-off and those with RC levels < cut-off was analysed using contingency tables and the chi-square test. Comparison of the cumulative survival rates between the two groups was performed using the Kaplan–Meier survival analysis with the log-rank test. The multivariable Cox hazard model using variables with statistical significance in the univariable Cox hazard model was used to obtain hazard ratios (HRs) during the considerable follow-up duration. *p* values < 0.05 were considered statistically significant.

## 3. Results

### 3.1. Characteristics of Patients with AAV at Diagnosis and During Follow-Up

Regarding the variables at AAV diagnosis, the median age of the 139 patients (male, 31.7%) was 58.0 years. The median BMI was within the normal range (22.0 kg/m^2^), and 10 patients had smoked cigarettes (no currently smoking patients). Of the 139 patients, 77, 30, and 32 were diagnosed with MPA, GPA, and EGPA, respectively. The median BVAS, FFS, ESR, and CRP levels were 13.0, 1.0, 60.5 mm/h, and 7.0 mg/L, respectively. The median total cholesterol, HDL-C, TG, and LDL-C levels were 171.0, 48.0, 108.0, and 96.8 mg/dL, respectively. The median RC level was 21.6 mg/dL. Hypertension and T2DM were present in 41 and 39 patients, respectively. Regarding the variables during follow-up after AAV diagnosis, 11 patients died during a median follow-up duration of 34.1 months. The causes of death in these 11 patients were infection (*n* = 8), diffuse alveolar haemorrhage (*n* = 1), interstitial lung disease (*n* = 1), and renal disease (*n* = 1). Furthermore, 11 patients experienced CVAs for a median follow-up duration of 31.5 months, and nine were diagnosed with ACS for a median follow-up duration of 32.9 months (Table 1).

### 3.2. AUC and Relative Risk for ACS

**Figure 1:**
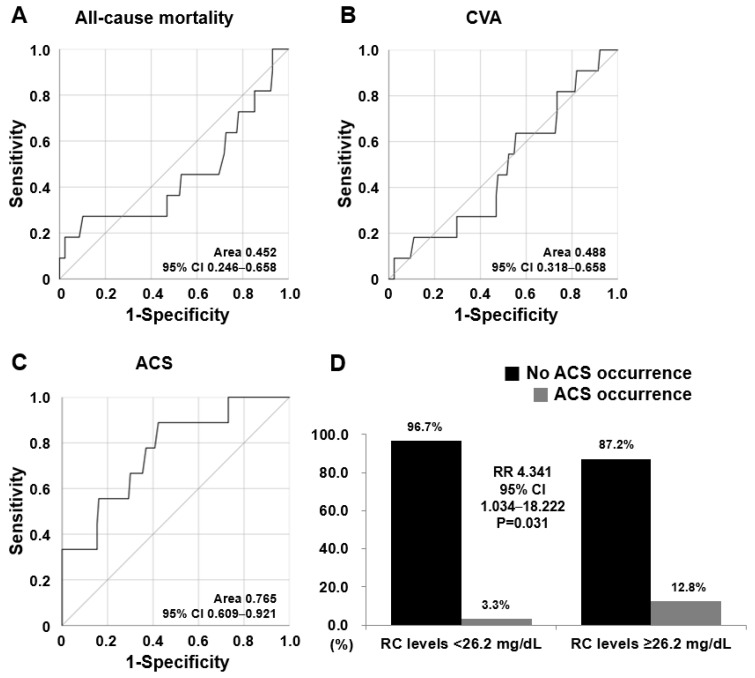
Area under the curve and relative risk for ACS. (**A**–**C**) Among the three poor outcomes, the AUC of RC levels for ACS was significant in the ROC curve analysis. (**D**) Relative risk of the cut-off of RC levels of 26.2 mg/dL for ACS occurrence was 4.341. ACS: acute coronary syndrome; AUC: area under the curve; CVA: cerebrovascular vascular accident; RC: remnant cholesterol; ROC: receiver operating characteristic.

Among the AUCs of RC levels for the three poor outcomes, only the AUC of RC levels for ACS were statistically significant (AUC 0.765, 95% confidence interval [CI] 0.609–0.921) (Figure 1A–C). When the optimal cut-off of RC levels for ACS was ≥26.2 mg/dL using the ROC curve, the sensitivity was 88.9% and the specificity was 57.7%. When we divided patients into two groups based on RC levels ≥26.2 mg/dL, ACS was identified more frequently in patients with RC levels ≥26.2 mg/dL than in those with RC levels <26.2 mg/dL (12.8% versus 3.3%, *p* = 0.031). Furthermore, patients with RC levels ≥26.2 mg/dL exhibited a significantly higher risk for ACS occurrence than did those with RC levels <26.2 mg/dL (RR 4.341, 95% CI 1.034–18.222) (Figure 1D). This result aligns with that when defining the highest tertile group based on 26.2 mg/dL, which is 66.6 percentile of the RC levels. Therefore, this result may support the validity of the methods used to divide patients with AAV into RC tertile groups and compare the clinical significance of RC levels.

### 3.3. Number of Patients with ACS in Each Tertile Group Based on RC Levels

**Figure 2:**
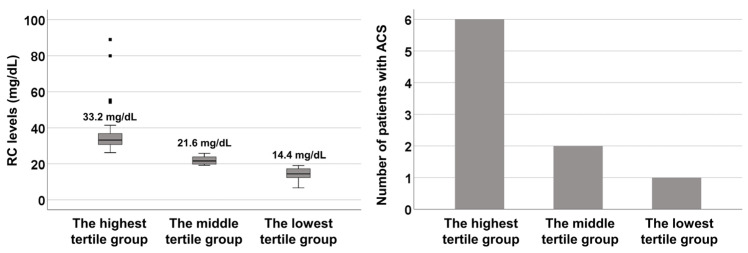
Tertile groups according to RC levels. The grey squares in the left panel represent the interquartile range. RC: remnant cholesterol; ACS: acute coronary syndrome.

The median RC levels in the highest, middle, and lowest tertile groups were 33.2, 21.6, and 14.4 mg/dL, respectively. During follow-up, six, two, and one patients were diagnosed with ACS in the highest, middle, and lowest tertile groups, respectively (Figure 2).

### 3.4. Comparison of ACS-Free Survival Rates

**Figure 3:**
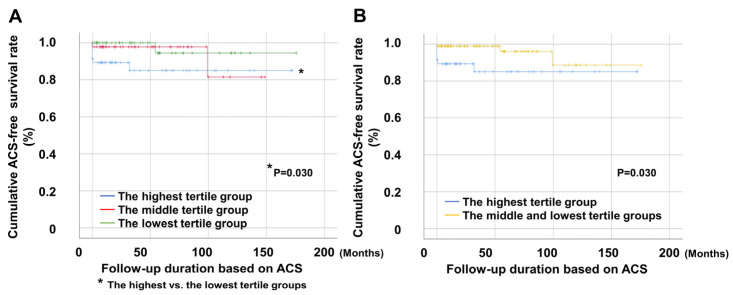
Comparison of ACS-free survival rates. (**A**) The highest tertile group had a significantly lower ACS-free survival rate than the lowest tertile group. (**B**) The highest tertile group had a significantly lower ACS-free survival rate than the middle and lowest tertile groups. ACS: acute coronary syndrome; RC: remnant cholesterol.

Patients in the highest tertile group showed significantly lower ACS-free survival rates than those in the lowest tertile. However, no significant differences were observed between the remaining groups (Figure 3A). Additionally, patients in the highest tertile group showed a significantly reduced ACS-free survival rate compared to those in the middle and lowest tertile groups (Figure 3B).

### 3.5. Cox Analyses

In the univariable Cox analysis, male sex, BVAS, T2DM, RC levels, and the highest tertile of RC levels at AAV diagnosis were significantly associated with ACS occurrence during follow-up in patients with AAV. Ex-smoking and FFS were also associated with ACS occurrence; however, this was not statistically significant. In the multivariable Cox analysis with RC levels, BVAS (HR 1.116, 95% CI 1.003–1.242) and RC levels (HR 1.054, 95% CI 1.023–1.085) at diagnosis were significantly and independently associated with ACS occurrence during follow-up in patients with AAV. Furthermore, in the multivariable Cox analysis with the highest tertile of RC levels, male sex (HR 9.054, 95% CI 1.786–45.910), BVAS (HR 1.147, 95% CI 1.033–1.274), and the highest tertile of RC levels (HR 10.818, 95% CI 1.867–62.689) were significantly and independently associated with ACS occurrence during follow-up in patients with AAV (Table 2).

## 4. Discussion

### 4.1. Summary

RC levels have been proposed as a major indicator of CVD occurrence not only in patients with high atherosclerotic CVD risk but also in the general population [24,25]. However, no studies have demonstrated an association between RC levels at AAV diagnosis and ACS during follow-up in patients with AAV. To the best of our knowledge, this is the first study to suggest a potential association. However, further studies with larger populations are needed to validate this association and elucidate the underlying pathophysiological mechanisms. We hypothesised two mechanisms by which RC levels at diagnosis could predict ACS occurrence during follow-up in patients with AAV.

### 4.2. Possible Mechanisms Underlying the Association

The first mechanism is not directly related to AAV and is a predictive mechanism for ACS based on the general atherosclerotic CVD induction mechanism of RC levels. RC refers to the cholesterol contained in TG-rich remnant lipoprotein particles, which are by-products of the metabolism of TG-rich lipoproteins (TRLs). Medium-sized TRLs may enter the arterial wall; however, they are much slower than LDL particles [26]. Moreover, saturated fatty acids and phospholipids containing oxidised fatty acids generated by the lipolysis of TRLs may be taken up by parenchymal cells to induce maladaptive inflammatory responses [27]. As the remnants are digested, TGs are degraded, while a large number of undigested cholesterol (RC) droplets remain in the macrophages [28].

Consequently, elevated plasma RC levels promote lipid infiltration into the arterial wall. After passing through the vascular endothelial layer, RC may accumulate and be absorbed by macrophages and smooth muscle cells, leading to foam cell formation and ultimately developing into atherosclerotic plaques. Moreover, RC may trigger platelet aggregation and thrombus formation by accelerating the formation of the prothrombinase complex and upregulating the expression of plasminogen activator inhibitor-1 [29]. Additionally, RC increases the production of reactive oxygen species and induces endothelial cell dysfunction [30]. Therefore, the atherogenic effects of RC may explain its association with increased incidence of ACS, as demonstrated in the present study.

The second mechanism is directly related to AAV and involves chronic inflammation-induced insulin resistance. Reportedly, chronic inflammation contributes to insulin resistance, even in patients without current metabolic syndrome [31]. Insulin resistance enhances the production of inflammatory cytokines and chemokines, resulting in a vicious cycle that exacerbates inflammation. Insulin resistance may also induce metabolic abnormalities by increasing TG and RC levels, provoking endothelial dysfunction, hypertension, and hypercoagulability, ultimately initiating CVD [32,33].

Meanwhile, in this study, vasculitis activity represented by BVAS at AAV diagnosis and only male sex among the traditional risk factors of CVD, along with RC levels, were significantly and independently associated with ACS occurrence during follow-up in patients with AAV (Table 2). Therefore, the proposed mechanisms can be summarised as follows: (i) during AAV diagnosis, high AAV activity may induce elevated inflammation-induced insulin resistance; (ii) insulin resistance may subsequently contribute to high TG levels, a feature of metabolic abnormalities, leading to high RC levels; (iii) persistent metabolic abnormalities may accelerate ACS occurrence during the entire follow-up period; and iv) male sex may act as a more critical risk factor for ACS than other traditional risk factors for CVD in patients with AAV (Figure S1). Therefore, we concluded that RC levels at diagnosis could independently predict the occurrence of ACS in patients with AAV, regardless of the traditional risk factors for CVD.

### 4.3. Interpretation of the ROC Curve and Cox Analyses

We used two statistical analytical methods to compare the abilities of RC, LDL-C, and HDL-C levels to predict ACS in 139 patients with AAV. When we performed the ROC curve analysis and compared the related AUC for ACS, we found that RC levels exhibited a significant AUC for ACS (AUC 0.765, *p* = 0.008). However, neither LDL-C nor HDL-C levels showed a significant AUC for ACS (Figure S2A). Additionally, univariable Cox hazards model analysis revealed that LDL-C (HR 1.010, 95% CI 0.997–1.024) and HDL-C (HR 0.968, 95% CI 0.930–1.007) levels were not significantly associated with ACS occurrence. Accordingly, LDL-C and HDL-C levels were not included in the multivariable Cox analysis.

Meanwhile, among the 139 patients with AAV, one patient had a TG level of 445 mg/dL, and it was strictly prohibited to estimate LDL-C levels using the Friedewald formula in patients with a TG level of >400 mg/dL. Thus, we excluded this patient and performed two statistical analyses using data from the remaining 138 patients with AAV. In the ROC curve analysis, RC levels exhibited a significant AUC for ACS (AUC 0.736, *p* = 0.026). LDL-C levels also exhibited a significant AUC for ACS (AUC 0.707, *p* = 0.050); however, this was not statistically significant. HDL-C levels showed no significant AUC for ACS (Figure S2B). In the univariable Cox analysis, RC levels (HR 1.069, 95% CI 1.031–1.108) were significantly associated with ACS occurrence. Conversely, neither LDL-C (HR 1.012, 95% CI 0.998–1.027) nor HDL-C (HR 0.977, 95% CI 0.938–1.018) levels were associated with ACS occurrence. Accordingly, LDL-C and HDL-C levels were not included in the multivariable Cox analysis. Overall, these results suggest that RC levels at diagnosis may be more useful in predicting ACS occurrence during follow-up than LDL-C or HDL-C levels in patients with AAV.

We conducted additional multivariable Cox analyses by including the variables with *p* < 0.1 in the univariable Cox analysis, ex-smoking status, and FFS, given the clinical significance of these variables (Table S1). In the multivariable analysis with RC levels, only RC levels (HR 1.055, 95% CI 1.019–1.092) were significantly and independently associated with ACS occurrence during follow-up in patients with AAV. In addition, in the multivariable analysis with the highest tertile of RC levels, male sex (HR 7.979, 95% CI 1.391–45.801), BVAS (HR 1.137, 95% CI 1.017–1.272), and the highest tertile of RC levels (HR 9.369, 95% CI 1.586–55.346) were significantly and independently associated with ACS occurrence during follow-up in patients with AAV. Given that the mean age of the study population was 58 years, at which women no longer benefit from protective cardiovascular factors, the association between male sex and ACS occurrence was intriguing. We speculate that the association between male sex and ACS occurrence may be attributed to smoking history. Among males, 10 patients had a history of smoking (ex-smokers), whereas no females had a history of smoking. This disparity in the proportion of ex-smokers between males and females may, at least partially, explain the observed association between male sex and ACS occurrence.

Furthermore, because age is a critical traditional risk factor for CVD [34], it was also included in another multivariable Cox analysis (Table S2) with ex-smoking status and FFS. Similarly, in the multivariable analysis with RC levels, only RC levels (HR 1.057, 95% CI 1.020–1.096) were significantly and independently associated with ACS during follow-up in patients with AAV. In addition, in the multivariate analysis of the highest tertile of RC, male sex (HR 8.533, 95% CI 1.468–49.607), BVAS (HR 1.132, 95% CI 1.013–1.265), and the highest tertile of RC levels (HR 10.524, 95% CI 1.620–68.383) were significantly and independently associated with ACS occurrence during follow-up in patients with AAV. As BMI shows a paradoxical U-shaped pattern with ACS and its cardiovascular outcomes [35], it was not included in the multivariable Cox analysis in this study. Therefore, we also concluded that RC levels at diagnosis could independently predict ACS occurrence in patients with AAV, regardless of the traditional risk factors of CVD.

### 4.4. Limitations

This study had some limitations. First, LDL-C levels were not directly measured at diagnosis in patients with AAV, and RC levels were calculated using the calculated LDL-C levels rather than the measured LDL-C levels. Therefore, we acknowledge that the results of the comparative analysis of the predictive abilities for ACS occurrence between RC and LDL-C levels may slightly differ from the actual results. However, the results of the Copenhagen General Population Study revealed a linear association between directly measured and calculated RC levels [36]. Therefore, the European Atherosclerosis Society recently suggested using calculated RC levels to assess the RC levels in individuals [37]. Furthermore, the limited sample size and retrospective study design restrict the generalisability of our findings. However, because ACS is a fatal clinical situation with few accurate predictive biomarkers in patients with AAV [38,39], this study serves as a pilot study to discover a novel biomarker for assessing the potential of ACS occurrence because it is the first to demonstrate that RC levels at diagnosis could predict ACS occurrence during follow-up. A future prospective study that includes more patients and measures total cholesterol, LDL-C, HDL-C, and TG levels directly and serially will provide more reliable and dynamic information on the predictive ability of RC levels for ACS occurrence in patients with AAV.

## 5. Conclusions

In this study, we demonstrated for the first time that RC levels at diagnosis may predict ACS occurrence during follow-up in patients with AAV, regardless of the traditional risk factors for CVD. Based on these results, we suggest that physicians must closely monitor RC levels at diagnosis, in addition to other lipid values, to better predict and manage ACS occurrence in patients newly diagnosed with AAV.

## Figures and Tables

**Table 1:** Characteristics of patients with AAV at diagnosis and during follow-up (*n* = 139).

Variables	Values
***At the AAV diagnosis***	
**Demographic data**	
Age (years)	58.0 (46.0–69.0)
Male sex (N, (%))	44 (31.7)
BMI (kg/m^2^)	22.0 (19.6–24.0)
Ex-smoker (N, (%))	10 (7.2)
**AAV subtype (N, (%))**	
MPA	77 (55.4)
GPA	30 (21.6)
EGPA	32 (23.0)
**ANCA type and positivity (N, (%))**	
MPO-ANCA (or P-ANCA) positivity	92 (66.2)
PR3-ANCA (or C-ANCA) positivity	22 (15.8)
Double ANCA positivity	5 (3.6)
**AAV-specific indices**	
BVAS	13.0 (7.0–18.8)
FFS	1.0 (1.0–2.0)
**Acute phase reactants**	
ESR (mm/hr)	60.5 (22.5–91.0)
CRP (mg/L)	7.0 (1.4–70.2)
**Lipid profile (mg/dL)**	
Total cholesterol	171.0 (145.0–202.0)
HDL-C	48.0 (36.0–63.0)
TG	108.0 (86.0–155.0)
LDL-C	96.8 (78.4–121.2)
**RC (mg/dL)**	21.6 (17.2–31.0)
**Comorbidities (N, (%))**	
Hypertension	41 (29.5)
T2DM	39 (28.1)
***During the follow-up duration***	
**Typical poor outcomes of AAV**	
All-cause mortality (N, (%))	11 (7.9)
Follow-up duration based on all-cause mortality (months)	34.1 (11.5–75.7)
CVA (N, (%))	11 (7.9)
Follow-up duration based on CVA (months)	31.5 (9.2–70.1)
ACS (N, (%))	9 (6.5)
Follow-up duration based on ACS (months)	32.9 (9.8–72.0)

Values are expressed as median (25–75 percentiles) or N (%). AAV: ANCA-associated vasculitis; ANCA: antineutrophil cytoplasmic antibody; BMI: body mass index; MPA: microscopic polyangiitis; GPA: granulomatosis with polyangiitis; EGPA: eosinophilic granulomatosis with polyangiitis; MPO: myeloperoxidase; P: perinuclear; PR3: proteinase 3; C: cytoplasmic; BVAS: Birmingham vasculitis activity score; FFS: five-factor score; ESR: erythrocyte sedimentation rate; CRP: C-reactive protein; HDL-C: high-density lipoprotein cholesterol; TG: triglyceride; LDL-C: low-density lipoprotein cholesterol; RC: remnant cholesterol; T2DM: type 2 diabetes mellitus; CVA: cerebrovascular vascular accident; ACS: acute coronary syndrome.

**Table 2:** Cox hazard model analyses of variables at diagnosis for ACS occurrence during follow-up in patients with AAV.

Variables	Univariable			Multivariable (RC Levels)			Multivariable (The Highest Tertile of RC Levels)		
	HR	95% CI	*p* Value	HR	95% CI	*p* Value	HR	95% CI	*p* Value
Age (years)	1.015	0.967–1.065	0.542						
Male sex (N, (%))	5.613	1.374–22.927	0.016	2.788	0.564–13.766	0.208	9.054	1.786–45.910	0.008
BMI (kg/m^2^)	1.131	0.922–1.388	0.238						
Ex-smoker (N, (%))	4.635	0.948–22.671	0.058						
MPO-ANCA (or P-ANCA) positivity	2.185	0.447–10.670	0.334						
PR3-ANCA (or C-ANCA) positivity	0.574	0.071–4.609	0.601						
BVAS	1.130	1.028–1.243	0.012	1.116	1.003–1.242	0.044	1.147	1.033–1.274	0.010
FFS	1.838	1.001–3.376	0.050						
ESR (mm/h)	1.006	0.989–1.023	0.514						
CRP (mg/L)	1.001	0.991–1.011	0.862						
Hypertension	2.604	0.692–9.801	0.157						
T2DM	5.139	1.285–20.554	0.021	3.782	0.904–15.818	0.068	4.057	0.927–17.752	0.063
RC levels (mg/dL)	1.065	1.036–1.095	<0.001	1.054	1.023–1.085	<0.001			
The highest tertile of RC levels	4.077	1.019–16.318	0.047				10.818	1.867–62.689	0.008

ACS: acute coronary syndrome; AAV: ANCA-associated vasculitis; ANCA: antineutrophil cytoplasmic antibody; BMI: body mass index; MPO: myeloperoxidase; P: perinuclear; PR3: proteinase 3; C: cytoplasmic; BVAS: Birmingham vasculitis activity score; FFS: five-factor score; ESR: erythrocyte sedimentation rate; CRP: C-reactive protein; T2DM: type 2 diabetes mellitus; RC: remnant cholesterol.

## Data Availability

The data presented in this study are available from the corresponding author upon request.

## Supplementary Materials

The following supporting information can be downloaded at: https://www.mdpi.com/article/10.3390/jcm14072260/s1, Table S1: Cox hazard model analyses of variables at diagnosis for ACS occurrence during follow-up in patients with AAV; Table S2: Cox hazard model analyses of variables at diagnosis for ACS occurrence during follow-up in patients with AAV; Figure S1: Proposed mechanism of the association between RC levels and ACS occurrence; Figure S2: Area under the curve of RC, LDL-C, and HDL-C levels for ACS in A) all patients and in B) excluding patients with a TG level >400 mg/dL.

## Abbreviations

The following abbreviations are used in this manuscript:

ANCA	antineutrophil cytoplasmic antibody
AAV	antineutrophil cytoplasmic antibody-associated vasculitis
MPA	microscopic polyangiitis
GPA	granulomatosis with polyangiitis
EGPA	eosinophilic granulomatosis with polyangiitis
CVD	cardiovascular disease
ACS	acute coronary syndrome
STEMI	ST-elevation myocardial infarction
LDL-C	low-density lipoprotein cholesterol
TG	triglyceride
RC	remnant cholesterol
SHAVE	Severance Hospital ANCA-associated Vasculitides
IRB	Institutional Review Board
BMI	body mass index
BVAS	Birmingham vasculitis activity score
FFS	five-factor score
P-ANCA	perinuclear-antineutrophil cytoplasmic antibody
C-ANCA	cytoplasmic-antineutrophil cytoplasmic antibody
MPO	myeloperoxidase
PR3	proteinase 3
ESR	erythrocyte sedimentation rate
CRP	C-reactive protein
HDL-C	high-density lipoprotein cholesterol
T2DM	type 2 diabetes mellitus
CVA	cerebrovascular accidents
ROC	receiver operating characteristic
ANOVA	analysis of variance
RR	relative risk
HRs	hazard ratios
CI	confidence interval
TRL	triglyceride-rich lipoproteins
