# Supplementary material for: Remnant Cholesterol Levels at Diagnosis May Predict Acute Coronary Syndrome Occurrence During Follow-Up in Patients with Antineutrophil Cytoplasmic Antibody-Associated Vasculitis
Source: J Clin Med. 2025 Mar 26;14(7):2260. doi: 10.3390/jcm14072260 (PMC11989813; doi:10.3390/jcm14072260)
Supplement: Supplementary file 1 [file jcm-14-02260-s001.zip › jcm-3537672-supplementary.pdf]

Supplementary Table S1. Cox hazards model analyses of variables at diagnosis for ACS during follow-up in AAV patients

Variables	Univariable			Multivariable (RC levels)			Multivariable (The highest tertile of RC levels)		
	**HR**	**95% CI**	**P value**	**HR**	**95% CI**	**P value**	**HR**	**95% CI**	**P value**
Age (years)	1.015	0.967-1.065	0.542						
Male sex (N, (%))	5.613	1.374-22.927	0.016	2.521	0.488-13.029	0.270	7.979	1.391-45.801	0.020
BMI (kg/m^2)	1.131	0.922-1.388	0.238						
Ex-smoker (N, (%))	4.635	0.948-22.671	0.058	1.565	0.177-13.814	0.687	1.479	0.240-9.101	0.673
MPO-ANCA (or P-ANCA) positivity	2.185	0.447-10.670	0.334						
PR3-ANCA (or C-ANCA) positivity	0.574	0.071-4.609	0.601						
BVAS	1.130	1.028-1.243	0.012	1.118	0.994-1.258	0.063	1.137	1.017-1.272	0.024
FFS	1.838	1.001-3.376	0.050	0.888	0.429-1.842	0.750	1.108	0.593-2.068	0.749
ESR (mm/hr)	1.006	0.989-1.023	0.514						
CRP (mg/L)	1.001	0.991-1.011	0.862						
Hypertension	2.604	0.692-9.801	0.157						
T2DM	5.139	1.285-20.554	0.021	4.700	0.845-26.130	0.077	3.955	0.854-18.308	0.079
**RC levels (mg/dL)**	1.065	1.036-1.095	<0.001	1.055	1.019-1.092	0.002			
**The highest tertile of RC levels**	4.077	1.019-16.318	0.047				9.369	1.586-55-346	0.014

ACS: acute coronary syndrome; AAV: ANCA-associated vasculitis; ANCA: antineutrophil cytoplasmic antibody; BMI: body mass index; MPO: myeloperoxidase; P: perinuclear; PR3: proteinase 3; C: cytoplasmic; BVAS: Birmingham vasculitis activity score; FFS: five-factor score; ESR: erythrocyte sedimentation rate; CRP: C-reactive protein; T2DM: type 2 diabetes mellitus; RC: remnant cholesterol.

Supplementary Table S2. Cox hazards model analyses of variables at diagnosis for ACS during follow-up in AAV patients

Variables	Univariable			Multivariable (RC levels)			Multivariable (The highest tertile of RC levels)		
	**HR**	**95% CI**	**P value**	**HR**	**95% CI**	**P value**	**HR**	**95% CI**	**P value**
Age (years)	1.015	0.967-1.065	0.542	0.959	0.886-1.038	0.298	0.962	0.891-1.038	0.318
Male sex (N, (%))	5.613	1.374-22.927	0.016	2.457	0.452-13.365	0.487	8.533	1.468-49.607	0.017
BMI (kg/m^2)	1.131	0.922-1.388	0.238						
Ex-smoker (N, (%))	4.635	0.948-22.671	0.058	2.266	0.226-22.757	0.487	1.783	0.275-11.567	0.545
MPO-ANCA (or P-ANCA) positivity	2.185	0.447-10.670	0.334						
PR3-ANCA (or C-ANCA) positivity	0.574	0.071-4.609	0.601						
BVAS	1.130	1.028-1.243	0.012	1.111	0.991-1.245	0.071	1.132	1.013-1.265	0.029
FFS	1.838	1.001-3.376	0.050	1.047	0.472-2.325	0.909	1.325	0.645-2.722	0.443
ESR (mm/hr)	1.006	0.989-1.023	0.514						
CRP (mg/L)	1.001	0.991-1.011	0.862						
Hypertension	2.604	0.692-9.801	0.157						
T2DM	5.139	1.285-20.554	0.021	7.464	0.916-60.782	0.060	5.620	0.940-33.589	0.058
**RC levels (mg/dL)**	1.065	1.036-1.095	<0.001	1.057	1.020-1.096	0.002			
**The highest tertile of RC levels**	4.077	1.019-16.318	0.047				10.524	1.620-68.383	0.014

ACS: acute coronary syndrome; AAV: ANCA-associated vasculitis; ANCA: antineutrophil cytoplasmic antibody; BMI: body mass index; MPO: myeloperoxidase; P: perinuclear; PR3: proteinase 3; C: cytoplasmic; BVAS: Birmingham vasculitis activity score; FFS: five-factor score; ESR: erythrocyte sedimentation rate; CRP: C-reactive protein; T2DM: type 2 diabetes mellitus; RC: remnant cholesterol.

Supplementary Figure S1. Proposed mechanism of association between RC levels and ACS occurrence
ACS: acute coronary syndrome; AAV: ANCA-associated vasculitis; BMI: body mass index; BVAS: Birmingham vasculitis activity score; T2DM: type 2 diabetes mellitus; RC: remnant cholesterol; TG: triglyceride.

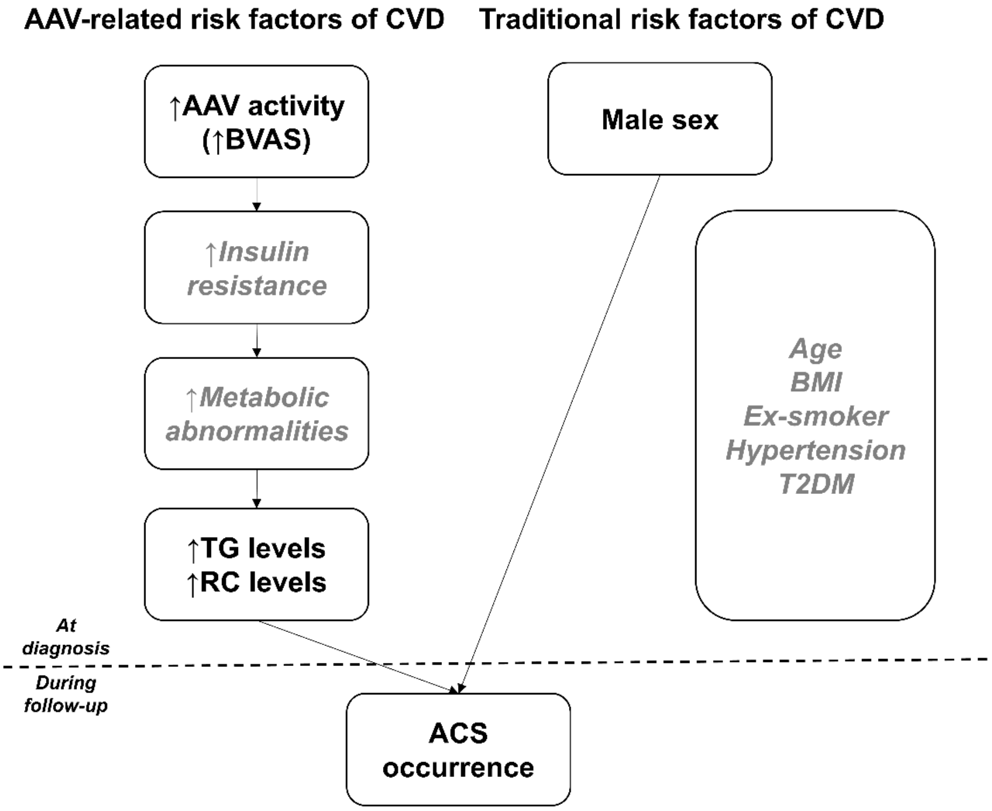

Supplementary Figure S2. Area under the curve of RC, LDL-C, and HDL-C levels for ACS in A) all patients, and in B) excluding patients with a TG level > 400 mg/dL

ACS: acute coronary syndrome; CI: confidence interval; HDL-C: high-density lipoprotein cholesterol; LDL-C: low-density lipoprotein cholesterol; TG: triglyceride.

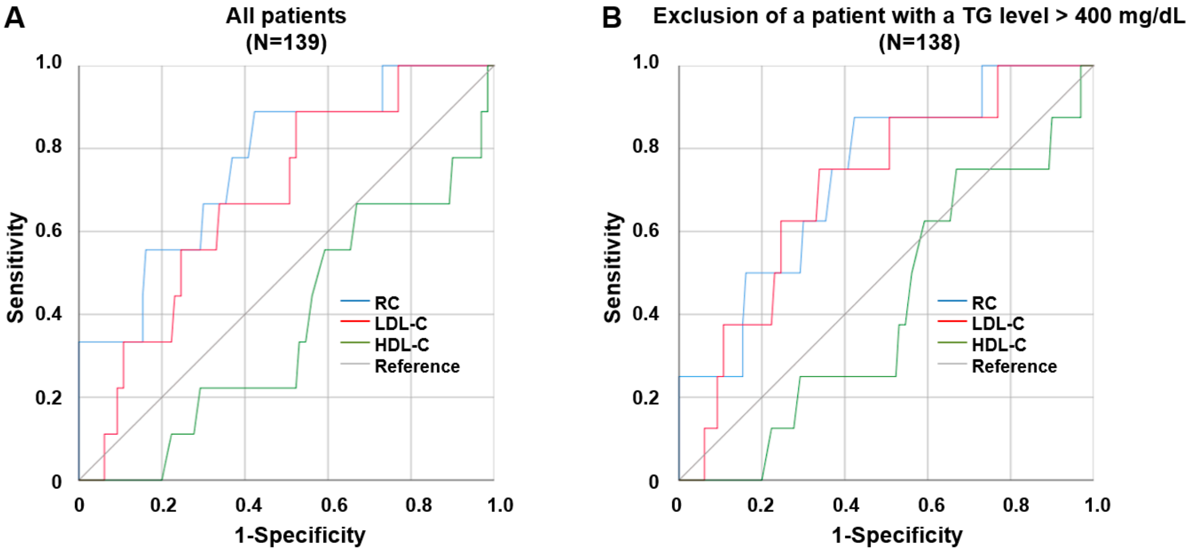

Variables	Area	P-value	95% CI
RC	0.765	0.008	0.609-0.921
LDL-C	0.681	0.070	0.524-0.838
HDL-C	0.371	0.195	0.190-0.551

Variables	Area	P-value	95% CI
RC	0.736	0.026	0.574-0.901
LDL-C	0.707	0.050	0.541-0.872
HDL-C	0.371	0.195	0.190-0.551
